# 1027. Case Study. A Novel Application of Blood Flow Restriction Therapy in a Burn Patient

**DOI:** 10.1093/jbcr/irag033.543

**Published:** 2026-04-06

**Authors:** Adam M Meyer, Deanna C Denman, Alicia M Williams

**Affiliations:** US Army Institute of Surgical Research Burn Center, Joint Base San Antonio, Fort Sam Houston, TX; US Army Institute of Surgical Research Burn Center, Joint Base San Antonio, Fort Sam Houston, TX; US Army Institute of Surgical Research Burn Center, Joint Base San Antonio, Fort Sam Houston, TX

## Abstract

**Patient Presentation (age range, injury details, relevant history):**

A 58-year-old male with 27.5% burns, primarily to both upper and lower extremities, presented to our Burn Clinic 7 months post injury with persistent open wounds to the lower extremities (see Figures).

**Clinical Challenges:**

Burn patients often face unique challenges, including loss of lean muscle mass, acute and chronic pain, and delayed wound healing. Blood flow restriction (BFR) has shown promise in improving strength and functional outcomes in hospitalized and critically ill patients. BFR is hypothesized to increase human growth factor expression and angiogenesis, potentially supporting improved healing in complex cases. While BFR has been used in trauma populations, it has not been widely applied in burn care. In a prior case at our burn center, BFR was associated with a faster rate of wound closure. Here, we present a novel application of BFR in a patient with chronic, non-healing wounds.

**Management Approach:**

BFR was applied to the right lower extremity using a passive approach protocol (80% occlusion for 5 minutes followed by 3 minutes of reperfusion for 3 rounds) for 8 sessions across 3 weeks. We collected pre- and post-treatment circumferential measurements of the thighs, assessed pain with the Defense/Veteran’s Pain Rating Scale, and estimated wound healing via serial photography. The left leg, which did not receive BFR, served as a comparison.

**Outcomes:**

The patient reported a reduction in pain intensity across treatment sessions (from 5/10 to 1/10). Limb circumference was maintained with no difference between limbs, suggesting preservation of muscle mass. Comparison across serial photographs revealed significantly greater wound healing in the BFR-treated limb. At the conclusion of treatment, the right lower extremity had completely epithelialized, while the left leg continued to exhibit open wounds. The patient reported a positive overall experience with BFR, noting rapid wound healing, decreased pain, and improved ability to return to physical activity.

**Lessons Learned:**

BFR may offer meaningful benefits in burn care by reducing pain, preserving muscle mass, and accelerating wound healing. This case supports the feasibility of BFR in outpatient burn rehabilitation and suggests further studies are warranted.

**Applicability to Practice:**

BFR therapy, already in use among critically ill patients and among amputees, may represent a valuable adjunct in burn rehabilitation—particularly for patients at risk of chronic wounds and muscle loss.

